# Size-dependent enhancement of gene expression by *Plasmodium* 5’UTR introns

**DOI:** 10.1186/s13071-024-06319-0

**Published:** 2024-05-27

**Authors:** Lirong Lin, Yanjing Liu, Rui Liang, Yue Guo, Ruixue Xu, Ruoxi Fan, Zhiwei Jiao, Wenting Zhao, Lixia Yue, Mingke Lu, Shengfa Liu, Xin-zhuan Su, Jian Li

**Affiliations:** 1 State Key Laboratory of Cellular Stress Biology, School of Life Sciences, Faculty of Medicine and Life Sciences, Xiamen University, Xiamen, 361102 Fujian China; 2 School of Medicine, Huzhou University, Huzhou, 313000 Zhejiang China; 3 Laboratory of Malaria and Vector Research, National Institute of Allergy and Infectious Diseases, National Institutes of Health, Rockville, MD 20850 USA

**Keywords:** *Plasmodium yoelii*, Untranslated region, Luciferase activity, CRISPR/Cas9, Gene expression regulation

## Abstract

**Background:**

Eukaryotic genes contain introns that are removed by the spliceosomal machinery during mRNA maturation. Introns impose a huge energetic burden on a cell; therefore, they must play an essential role in maintaining genome stability and/or regulating gene expression. Many genes (> 50%) in *Plasmodium* parasites contain predicted introns, including introns in 5′ and 3′ untranslated regions (UTR). However, the roles of UTR introns in the gene expression of malaria parasites remain unknown.

**Methods:**

In this study, an episomal dual-luciferase assay was developed to evaluate gene expression driven by promoters with or without a 5′UTR intron from four *Plasmodium yoelii* genes. To investigate the effect of the 5′UTR intron on endogenous gene expression, the *pytctp* gene was tagged with 3xHA at the N-terminal of the coding region, and parasites with or without the 5′UTR intron were generated using the CRISPR/Cas9 system.

**Results:**

We showed that promoters with 5′UTR introns had higher activities in driving gene expression than those without 5′UTR introns. The results were confirmed in recombinant parasites expressing an HA-tagged gene (*pytctp*) driven by promoter with or without 5′UTR intron. The enhancement of gene expression was intron size dependent, but not the DNA sequence, e.g. the longer the intron, the higher levels of expression. Similar results were observed when a promoter from one strain of *P. yoelii* was introduced into different parasite strains. Finally, the 5′UTR introns were alternatively spliced in different parasite development stages, suggesting an active mechanism employed by the parasites to regulate gene expression in various developmental stages.

**Conclusions:**

*Plasmodium* 5′UTR introns enhance gene expression in a size-dependent manner; the presence of alternatively spliced mRNAs in different parasite developmental stages suggests that alternative slicing of 5′UTR introns is one of the key mechanisms in regulating parasite gene expression and differentiation.

**Graphical Abstract:**

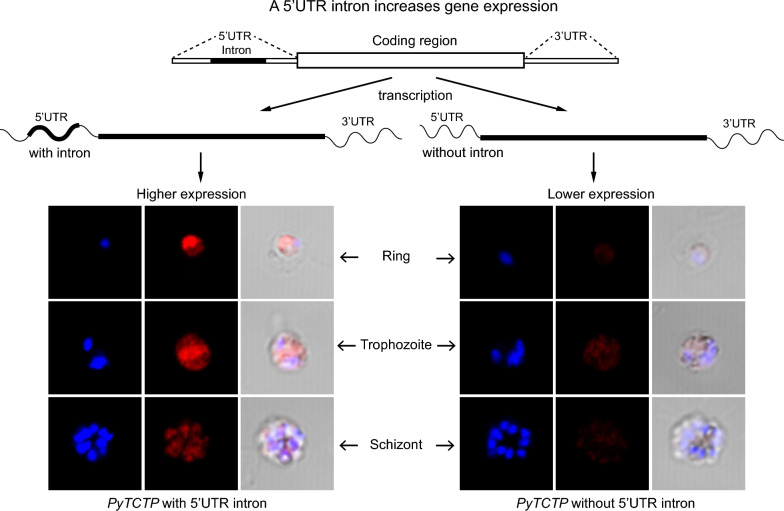

**Supplementary Information:**

The online version contains supplementary material available at 10.1186/s13071-024-06319-0.

## Background

Malaria is one of the major infectious diseases that affects hundreds of millions of people worldwide and kills over 500,000 people annually [1]. Malaria parasites have a complex life cycle in which sexual and asexual reproduction occurs alternately between female *Anopheles* mosquitoes and vertebrate hosts. The complicated regulation of gene expression in different developmental stages of the *Plasmodium* life cycle is critical for the parasites to adapt to different environments [2–4]. Gene expressions in *Plasmodium* parasites are regulated by various mechanisms, including transcription factors, unique promoters, enhancers/inhibitors, non-coding RNA, codon usage bias, chromatin structure and various epigenetic modifications on nucleic acids and histone proteins [5–11]. Many genes in *Plasmodium* parasites are interrupted by one or more introns [12–15], which may impose an energetic burden on a cell [16]; however, studies on the roles of introns in gene expression regulation of the parasites are limited. In the human malaria parasite *Plasmodium falciparum*, the *var* gene introns were shown to have bi-directional promoter activities driving the transcription of longer non-coding RNAs (lnc-RNAs) as antisense of exon 1 and sense in exon 2 [17–20]. lncRNA can act *in cis* or *in trans* to recruit chromatin-modifying complexes to the targeted loci to generate repressive heterochromatin. The bi-directional promoter activity within the *var* intron is vital for silencing the upstream *var* promoter [17, 18]. However, whether the presence of an intron or the size of an intron can affect gene expression in malaria parasites, particularly introns in 5′ and 3′ untranslated regions (UTRs), remains unknown.

In a eukaryotic cell, introns have been shown to regulate gene expression through various mechanisms [21–23]. Introns can act as a repressor, a transcriptional enhancer, or an element maintaining locus-specific chromatin opening state [24–26]. Introns can enhance the export of mRNAs via the nuclear pore complex (NPC) by recruiting export factors to spliced RNAs as part of the exon junction complex [27–30]. An intron can also influence translation efficiency, which is highly dependent on the position of an intron [31, 32]. Introns can increase alcohol dehydrogenase-1 (*adhl*) gene expression in maize, and the optimal location for such an intron enhancement is near the 5' end of the mRNA [21]. Additionally, the size of an intron also influences gene expression; the longer an intron, the greater the relative level of gene expression [33].

In addition, within the coding sequence, introns can be found at 5′ and 3′ UTRs of a gene, and various studies have shown that these UTR introns play an essential role in regulating gene expression [25, 33, 34]. In *Arabidopsis thaliana*, it was found that the density of 5'UTR introns was significantly higher than that of 3'UTRs (but similar to those of introns in coding regions); introns were longer in the coding regions and 3'UTRs and had a different nucleotide composition from those of coding regions and 3'UTR introns [33]. A 5′UTR intron of the polyubiquitin gene (*rubi3*) was found to elevate the beta-glucuronidase (GUS) mRNA expression level and to stimulate the tissue-specific enhancement of translation [12]. Interestingly, an intron placed in the 5′UTR could promote translation, but it became repressive when placed at the 3′UTR of the gene [32].

In one of our previous studies, we identified > 300 5′ and 3′UTR introns from the *Plasmodium yoelii* parasite [15]. Like those found in *A. thaliana*, approximately threefold more 5’UTR introns than 3’UTR introns were observed in the *P. yoelii* genes. Because these introns are not in an amino acid coding region, they likely function to regulate the gene expression of the parasites. Here, we designed experiments to investigate the role of selected *P. yoelii* 5′UTR introns in regulating gene expression. Using an episomal dual-luciferase cassette and parasites with CRISPR/Cas9-mediated gene knock-in and epitope tagging, we show that *P. yoelii* 5′UTR introns can significantly enhance gene expression. The regulatory activity of an intron is dependent on intron size but independent of DNA sequence. This study demonstrates that 5′UTR introns play an essential role in the regulation of malaria parasite gene expression and likely in parasite development.

## Methods

### Malaria parasites, mice and mosquitoes

The *P. yoelii* 17XL, BY265 and NSM parasites used in this study were described previously [35]. Female outbred ICR mice and inbred BALB/c mice, 6–8 weeks old, used to maintain parasites and evaluate parasite growth, respectively, were purchased from Xiamen University Laboratory Animal Center or Shanghai Laboratory Animal Center, CAS (SLACCAS). A colony of *Anopheles stephensi* mosquitoes (Hor strain) was raised at 23 °C and 75% humidity under a 12:12 light-dark illumination cycle and fed with 5% sucrose solution.

### Plasmid construction and transfection

**Fig. 1:**
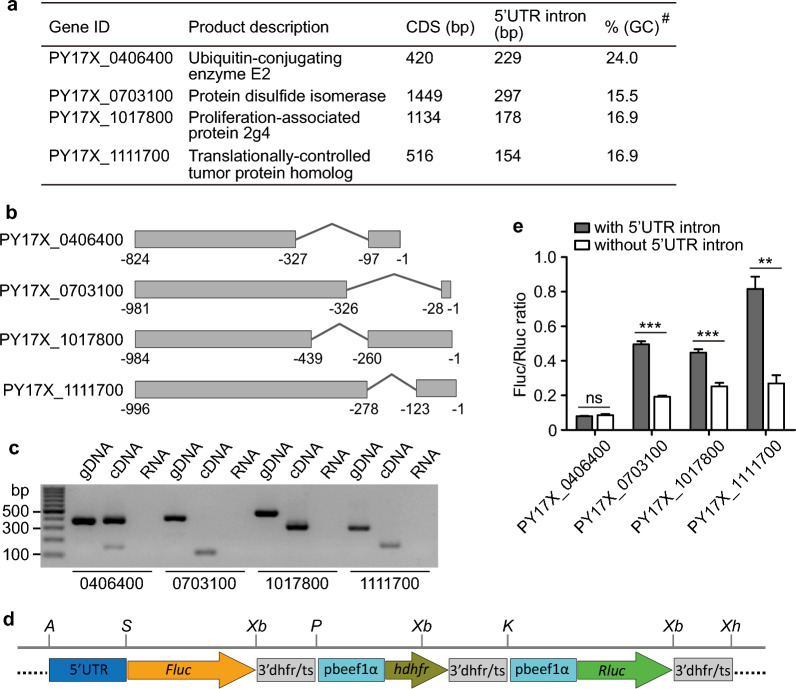
Dual-luciferase assay measuring promoter activities with or without 5’UTR introns. **a** Four *Plasmodium yoelii* genes having 5’UTR introns were tested in this study. #, the percentage of guanine/cytosine in the sequence of 5’UTR intron. **b** Diagrams showing positions and relative sizes of 5’UTR introns and the length of putative promoters used. The exact sizes of the putative promoters are unknown; the coding region starts at the position after ‘-1’ to the right. **c** Agarose gel of PCR products amplified from asexual blood stages of *P. yoelii* 17XL genomic DNA (gDNA), complementary DNA (cDNA), or RNA (without reverse transcriptase as a control for DNA contamination) using specific primer pairs -F1 and -R1 for each gene (Table S1). **d** Diagram of a plasmid construct for the luciferase assay. The 5’UTR is the region to be tested; *Fluc*, the gene encoding firefly luciferase; 3’dhfr/ts, 3’UTR of *Plasmodium berghei* dihydrofolate reductase-thymidylate synthase; pbeef1α, *P. berghei* eEF1A promoter; *hdhfr*, gene encoding human dihydrofolate reductase; *Rluc*, gene encoding Renilla luciferase; restriction sites: Apa I (*A*); Sac II (*S*); Xba I (*Xb*); Pst I (*P*); Kpn I (*K*); Xho I (*Xh*). **e** Relative luciferase signals after transfection with the promoter regions with or without 5’UTR introns from four genes. Two-tailed *t*-test; **P < 0.01; ***P < 0.001

To study the effect of 5′UTR intron on gene expression, we constructed a vector expressing dual luciferases that contain two reporter genes—Fluc and Rluc (Fig. 1)—based on pL0006 obtained from the Malaria Research and Reference Reagent Resource Center (MR4) (http://www.mr4.org/). In the first expression cassette, the putative parasite promoter regions (~ 0.6–1 kb) with or without 5’UTR intron were inserted into the vector at the 5′ of the Fluc gene as a transcriptional fusion. Thus, the effect of 5′UTR intron on transcription can be evaluated by comparing the expression levels of the firefly luciferase with or without the 5′UTR intron. A Rluc gene under the control of a *pbeef1αa* promoter and *pbdhfr/ts* 3′UTR was also included as an internal control for plasmid copy numbers. The constructs were introduced into *P. yoelii* lines using electroporation, and the transfected parasites were injected intravenously (i.v., in 100 µl PBS) into mice. The injected animals were treated with pyrimethamine (7 mg/ml) for 4–7 days. Surviving parasites were harvested from the mouse blood when parasitemia reached 1–5%, and the luciferase activities were determined using a dual-luciferase reporter gene assay kit (E1910, Promega).

The methods of gene tagging and gene disruption using the CRISPR/Cas9 system were basically as described previously [36]. To construct the plasmids for tagging PyTCTP with 3 × HA at the N-terminal of the protein and simultaneously generated parasites with or without the 5′UTR intron, we first amplified a ~ 600-bp segment from the 5’UTR region with or without intron as left homologous arm and a 200–400-bp fragment from N-terminal coding region as right homologous arm using the primers in Table S1. A DNA sequence encoding the 3 × HA (YPYDVPDYAGAYPYDVPDYAGAYPYDVPDYA) was inserted between the left and right arms in the frame (Fig. 3a, b) and cloned into the pYC5 vector using restriction enzymes Nco I and Sac II.

To construct the plasmid to delete the coding region of the *pytctp* gene, we used primer pairs K55/K53 and K35/K33 to amplify 5’UTR and 3’UTR regions (~ 500 bp) as left and right homologous arms and cloned them into restriction sites of Kpn I/Nco I and Xho I/Eco RI of the pYC5 vector. Oligonucleotide sequences for sgRNAs (Table S1) designed to target specific sites in the 17XL genome were ligated into the plasmid using enzyme Bsm BI. All oligonucleotides were commercially synthesized by Xiamen Borui Biological Technology Co., Ltd. Transfection, drug selection of transformed parasites and limiting-dilution cloning were performed as described [36]. Integration events in the transformed parasites were first detected using PCR amplification and then confirmed by DNA sequencing.

### Phenotypic characterization of parasite

Growth rates and host mortality rates of genetically modified parasite clones, as well as the wild-type (WT) *P. yoelii* 17XL, were evaluated in female BALB/c mice (4 mice per single parasite clone). Each mouse was injected i.v. with an inoculum containing 1 × 10^6^ iRBCs. Giemsa-stained thin blood films were made daily from day 2 post-infection (pi). Parasitemias were measured by direct microscopic examination and recorded in Excel.

For gametocyte induction, ICR mice were pretreated with phenylhydrazine (PHZ, 20 mg/kg) via intraperitoneal injection, and mice were infected i.v. with 1 × 10^5^ iRBCs 3 days after the injection of PHZ. Gametocytes were counted using Giemsa-stained thin blood smears on day 4 after the infection. Gametocytemias were calculated as the proportion of gametocytes over iRBCs.

For blood feeding of mosquitoes, each recipient ICR mouse was injected i.v. with 1 × 10^6^ iRBCs. Approximately 100 female mosquitoes were allowed to feed on an infected mouse for 30 min on days 3–4 pi. Mosquito midguts were dissected, and oocysts were counted on day 8 after the blood feeding. Salivary glands were dissected on days 16–18 and harvested for homogenization, and the number of released sporozoites was counted in a hemocytometer.

### Immunofluorescence assay (IFA)

iRBCs were fixed using 4% paraformaldehyde and applied to a poly-l-lysine pre-treated coverslip. The fixed cells were permeabilized with 0.1% Triton X-100 in PBS for 10 min, blocked with 5% BSA/PBS for 60 min at room temperature and incubated with rabbit anti-HA mAb (1:2,000, Cell Signaling Technology) diluted in 5% BSA at 4℃ overnight. The samples were then incubated with goat-anti-rabbit antibody conjugated with Alexa Fluor 555 (1:2,000, Invitrogen) for 1 h. Cell nuclei were stained with Hoechst 33342 (1:2,000, ThermoFisher Scientific), mounted in 90% glycerol solution and sealed with nail polish. Images were captured and processed using the same settings on a Zeiss LSM 780 confocal microscope.

### Quantitative reverse-transcription PCR (qRT-PCR)

Total RNA was prepared from iRBCs using a Trizol reagent (15,596,018, Invitrogen) and then treated with DNase to remove genomic DNA contamination. cDNA was synthesized using 5 × All-In-One MasterMix (G492, abm). The real-time quantitative RCR was performed using *TransStart* Top Green qPCR SuperMix (AQ131, Transgen) in the CFX96 Touch qPCR System (Bio-Rad). The PCR thermal cycling started with 30 s at 94 ℃ for denaturation, followed by 40 cycles of 5 s at 94 ℃ and 30 s at 60 ℃. Gene expression changes were quantified using the ^∆∆^Ct method. *Actin I* (PY17X_1461900) was used as the endogenous control, and the primers are shown in Table S1.

## Results

### Gene expression cassette and effects of 5’UTR intron on gene expression

To investigate whether the UTR introns of malaria parasites can affect gene expression, we randomly selected four genes that have 5’UTR introns (PY17X_0406400, PY17X_0703100, PY17X_1017800 and PY17X_1111700) based on our previous observations [15] for this study (Fig. 1a, 1b). We amplified a segment of 5’UTR from each gene with (DNA as template) or without (cDNA as template) the 5’UTR intron from *P. y. yoelii* 17XL parasite using primers that also introduced Apa I and Sac II restriction sites. PCR products of expected sizes were obtained (Fig. 1c). We next constructed a vector based on plasmid pL0006 (MR4, deposited by Waters et al.) after introducing a cassette encoding pbeef1αa-*Rluc*-3’pbdhfr/ts through KpnI and XhoI sites and a DNA segment containing firefly luciferase (Fluc) gene and 3’pbdhfr/ts through SacII and PstI sites to express both Renilla luciferase (Rluc) and firefly luciferase simultaneously (Fig. 1d). The amplified UTR products with or without the 5′UTR intron from each gene were then cloned into the vector (5’UTR in Fig. 1d) and used to transfect *P. y. yoelii* 17XL parasite using electroporation. Transfected parasites were injected (i.v.) into mice and were fed with water containing pyrimethamine (7 mg/ml). After parasitemia reached 1–5%, blood samples were taken, and luciferase activities (fluorescent signals) were measured using a commercial kit (Promega). Fluc/Rluc fluorescent signal ratios from repeated experiments were obtained and plotted (the Rluc signals were used to adjust gene expression variation due to differences in plasmid copy numbers or other unknown factors). We observed significantly higher signals from constructs with 5’UTR intron in three of the four genes tested (except PY17X_0406400) (Fig. 1e). These results show that, in most cases, 5’UTR introns of malaria parasites can increase gene expression, similar to those reported in other organisms [25, 33].

### Effects of 5’UTR intron on gene expression in different *P. yoelii* strains

**Fig. 2:**
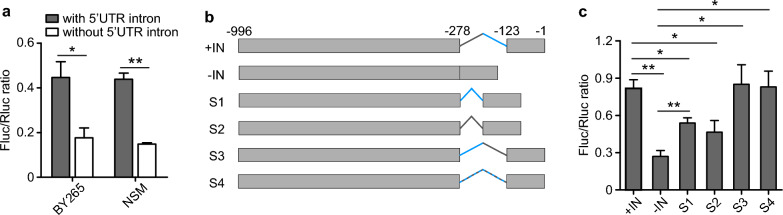
Activities of 5’UTR in different parasite strains and the effects of intron size and sequence on gene expression. **a** Relative luciferase signals after transfection of two different parasite strains (subspecies) with the promoter region of the PY17X_1111700 gene from *Plasmodium yoelii* 17XL with or without 5’UTR intron. **b** Diagrams showing PY17X_1111700 promoter regions with or without the original 5’UTR intron, half-size introns (S1 and S2), reversed intron sequence (S3) or an artificial sequence (S4). **c** Relative luciferase signals after transfection of *P. yoelii* 17XL parasites with plasmid constructs in **b**. Unpaired two-tailed *t*-test; *P < 0.05; **P < 0.01

We next investigated whether the same results could be obtained from different *P. yoelii* strains. We introduced the plasmids containing 5’UTR from PY17X_1111700 [translationally-controlled tumor protein (TCTP), also known as histamine-releasing factor (HRF)] with or without the 5’UTR intron into two additional parasites, *P. y. yoelii* BY265 and *P. y. nigeriensis* NSM (a subspecies), and obtained similar results as observed in the *P. y. yoelii* 17XL parasite, e.g. higher luciferase signals in parasites receiving promoter region with 5’UTR intron than those without the 5’UTR (Fig. 2a). The results suggest a similar mechanism in regulating gene expression levels in all *P. yoelii* strains or subspecies. The signals from the NSM and BY265 parasites having promoter sequence with 5’UTR intron were significantly lower than those of 17XL (*P* < 0.05), although the 5’UTR sequences from the three parasites had ~ 97% sequence similarity (Fig. S1). The results suggest that sequence variation in the intron and/or 5’UTR region flanking the intron can affect gene expression.

### Effects of 5’UTR intron size and sequence composition on gene expression

We next constructed plasmids containing *pytctp* 5’UTR region with modified intron sequences: type 1 and 2, smaller intron sequence containing the first or the second half of the original sequence, respectively (Fig. 2b); type 3, inverted sequence of the original intron; type 4, scrambled DNA sequence. We then evaluated the effects of these artificial sequences on gene expression. The parasites transfected with the smaller introns had significantly lower signal ratios than those transfected with its full-length 5′UTR intron (Fig. 2c). The parasites with inverted intron sequence or scrambled sequence had signal ratios similar to that of the original full-length intron (Fig. 2c). These results suggest that the size of the intron, but not the intron sequence, can influence gene expression.

### CRISPR/Cas9 modifications of *pytctp* 5’UTR intron and gene tagging

To further characterize the role of 5′UTR in mRNA and protein expression in the endogenous locus, we used CRISPR/Cas9 to tag the *pytctp* with 3xHA at the 5′ end of the gene and at the same time generated parasites with or without the 5′UTR intron. Using the *P. yoelii* 17XL parasite carrying the 5’UTR intron, we designed a guide RNA (GTCTTTATATACTTTCATTTTGG) at the 5′ region to insert the 3xHA tag to generate 5′ HA-tagged *pytctp* gene with or without the 5′UTR intron (Fig. S2a and S2b). After transfection of the 17XL parasites, pyrimethamine selection (7 mg/ml in drinking water) and limiting dilution cloning, we obtained parasite clones with expected insertions as determined using sequence-specific PCR (Fig. S2c and S2d). Correct 3xHA tagging of the gene was confirmed after DNA sequencing (Fig. S2e and S2f). Three parasite clones carrying HA-tagged gene with 5’UTR intron (*pytctp*^+IN+HA^ c1-3), three clones carrying HA-tag without the 5’UTR intron (*pytctp*^−IN+HA^ c1-3) and two un-tagged clones without the 5’UTR intron (*pytctp*^−IN^ c1-2) were eventually obtained. We also generated two parasite clones (*pytctp*^+IN+HAc^ c1-2) with a 3xHA-tag at the C-terminus of the PyTCTP protein similarly (Fig. S3a-3c).

### PyTCTP mRNA and protein expressions in parasites with or without 5’UTR intron

**Fig. 3:**
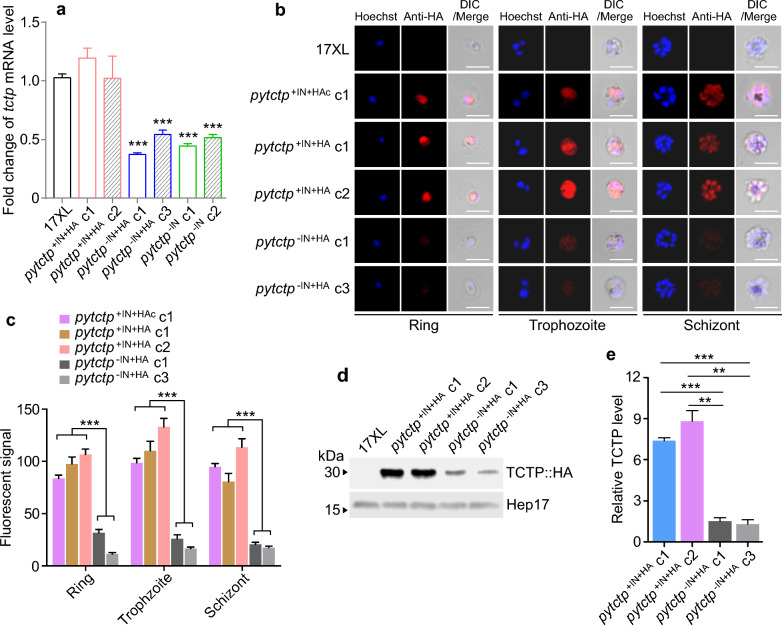
*pytctp* mRNA expression and PyTCTP protein expression levels in parasites with or without 5’UTR intron. **a** Quantitative RT-PCR analysis of *pytctp* gene expression. *pytctp*^+IN+HA^ c1 and *pytctp*^+IN+HA^ c2, two parasite clones with 5’UTR intron tagged with 3xHA at the N-terminus; *pytctp*^−IN+HA^ c1 and *pytctp*^−IN+HA^ c3, two N-terminus HA-tagged parasite clones without the 5’UTR intron; *pytctp*^−IN^ c1 and *pytctp*.^−IN^ c2, two parasite clones without the 5’UTR intron and no HA-tag. **b** Images of Immunofluorescence assay (IFA) of parasites (ring, trophozoite or schizont) expressing PyTCTP protein (red) stained by anti-HA antibody. *pytctp*^+IN+HAc^ c1, a parasite clone with 5’UTR intron tagged with 3xHA at the C-terminus. Hoechst 33,342 stain (blue) for nucleic acid. **c** Mean PyTCTP fluorescent signals of ring, trophozoite and schizont stages of the recombinant parasite clones. Signals were from 20 iRBC cells. **d** Western blot detection of HA-tagged PyTCTP protein in 17XL WT and HA-tagged parasite clones with or without 5’UTR intron. Hep17 (*P. yoelii* hepatocyte erythrocyte protein 17 kDa) as a protein loading control. **e** Mean fluorescent signals from protein gels as in **c** (from three independent experiments). For **a**, **c** and **e**, unpaired two-tailed *t*-test; **P < 0.01; ***P < 0.001

To investigate whether the 5’UTR intron could affect the levels of transcription or translation of endogenous genes in parasites, we used qPCR to measure the mRNA transcript of the *pytctp* gene from the genetically modified parasite clones. As expected, the parasites without the 5’UTR intron (*pytctp*^−IN+HA^ and *pytctp*^−IN^) had lower mRNA levels than those of WT 17XL, while the transcript levels of parasite clones carrying HA-tagged gene with 5′UTR intron (*pytctp*^+IN+HA^) were comparable to those of the WT parasite (Fig. 3a). These data indicate that the 5′UTR intron of the *pytctp* gene can enhance its transcriptional expression in vivo.

Next, we investigate PyTCTP protein expression using an anti-HA antibody. We performed immunofluorescence assay (IFA) on parasites with or without the 5′UTR intron (Fig. 3b). The PyTCTP protein was expressed at higher levels in the parasites with 5’UTR intron than in those without 5’UTR intron in ring, trophozoite and schizont stages with HA-tag at either N- or C-terminal (Fig. 3b). Quantification of fluorescent signals from randomly selected iRBCs (20 cells) showed significantly higher signals in parasites with 5’UTR intron than in those without the intron (Fig. 3c). The HA-tagged PyTCTP protein expression patterns suggest that it is expressed in the cytoplasm. The results show that the 5’UTR intron affects the *tctp* gene transcription and protein level but not the localization of the HA-tagged protein.

Next, we extracted parasite lysates from iRBCs after removing host white cells and performed a Western blot analysis using an anti-HA antibody. Western blot signals were detected using electrochemiluminescence (ECL) after incubation with anti-HA and horseradish peroxide-conjugated secondary antibodies. Stronger signals were detected in lysates from parasites carrying the *pytctp* gene with the 5’UTR intron than in those without the intron (Fig. 3d). Quantification of the protein signals showed significantly higher expression in parasites with the 5′UTR intron after adjusting for protein loading based on signal from anti-Hep17 (Fig. 3e). These results further confirm that the 5′UTR intron of the *pytctp* gene can increase its protein expression, which is consistent with those from episomal luciferase assays.

### Alternatively transcribed mRNAs in different parasite development stages

**Fig. 4:**
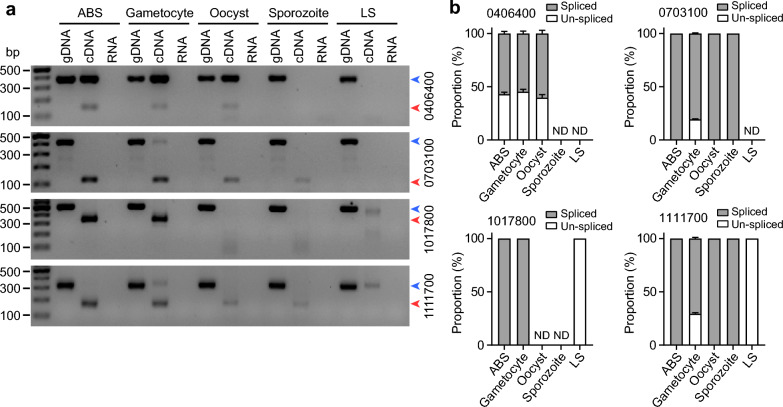
PCR analysis of alternatively spliced 5’UTR introns. **a** Agarose gel images showing PCR products amplified from DNA and cDNA isolated from various parasite developmental stages. ABS, asexual blood stages; LS, liver stages; gDNA, genomic DNA; cDNA, complementary DNA. The gene ID numbers are listed on the right side of the gels; bp, base pair. The blue and red arrowheads in each panel point to un-spliced and spliced variants, respectively. **b** Plots of the relative ratios (mean + SEM, four experimental repeats) of band signal from spliced form over the band from un-spliced form in **a**. The relative ratios of band signals were normalized to the size of DNA fragments in each plot. ND, no PCR product was amplified from the respective stages

The widespread presence of 5′ and 3′UTR introns in the parasite genome and our data showing increased gene expression with a 5′UTR intron suggest that the UTR introns could be alternatively spliced during parasite development in vertebrate and insect hosts to regulate gene expression. We next designed primers (Table S1) across the 5′UTR introns in the four genes and amplified cDNA prepared from blood stages, gametocytes, oocysts, sporozoites and liver stages. Alternative splicing events were observed for each of the four genes (Fig. 4a, b). For example, the 5’UTR intron of PY17X_1111700 (*pytctp*) was spliced in the blood stages, oocysts and sporozoites, and transcripts with the 5’UTR intron were observed in gametocytes and liver stages, suggesting increased gene expression in the two stages. These results suggest that alternative splicing of 5’UTR introns plays an essential role in parasite gene expression and parasite development and differentiation.

### Effects of 5’UTR intron or HA-tag on parasite growth, host survival and development of sexual stages

**Fig. 5:**
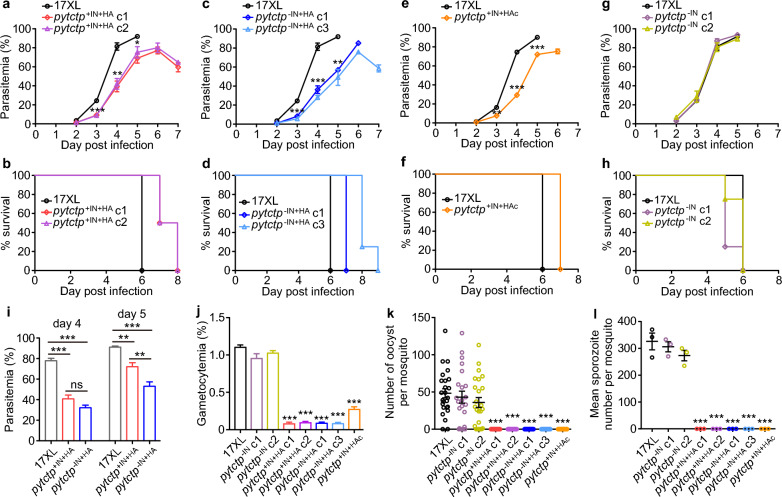
Parasitemia, host survival, gametocyte production, oocyst and sporozoite counts of 17XL and HA-tagged parasites with or without 5’UTR intron. **a** and **b** Parasitemia (**a**) and host survival (**b**) of 17XL wild type (WT) and two *pytctp*::HA parasite clones with 5’UTR intron. **c** and **d** Parasitemia (**c**) and host survival (**d**) of 17XL WT and two *pytctp*::HA parasite clones without 5’UTR intron. **e** and **f** Parasitemia (**e**) and host survival (**f**) of 17XL WT and the *pytctp* 3’ tagged parasite clone. **g** and **h** Parasitemia (**g**) and host survival (**h**) of 17XL WT and two *pytctp* un-tagged parasite clones without 5’UTR intron. **i** Days 4 and 5 parasitemia of the 17XL WT and *pytctp*::HA parasites with and without 5’UTR intron. **j**-**l** Gametocytemia (**j**), oocyst count (**k**) and sporozoite count (**l**) of 17XL WT, two *pytctp*::HA parasite clones with 5’UTR intron, two *pytctp*::HA parasite clones without 5’UTR intron, two *pytctp* un-tagged parasite clones without 5’UTR intron and one *pytctp* 3’ HA-tagged parasite clone. Unpaired two-tailed *t*-test; *P < 0.05; **P < 0.01; ***P < 0.001

We next evaluated the effects of the 5′UTR intron and the 3xHA-tag on parasite growth and host survival. Mice (four mice in each group) were injected with (1 × 10^6^ iRBCs, i.v.) WT or 3xHA-tagged 17XL parasites with or without the 5′UTR, and parasitemia and host survival were monitored daily. The parasites with the 3xHA-tag grew significantly more slowly and survived 1–2 days longer than the WT parasites (Fig. 5a–e), suggesting that the introduction of the 3xHA-tag may affect parasite growth. However, the absence of the 5’UTR intron did not significantly affect parasite growth in mice or host mortality (Fig. 5g, h), likely because of the splicing of the 5’UTR intron in the asexual stages of the 17XL parasites. Interestingly, the *pytctp*^+IN+HA^ parasites grew significantly more quickly than the *pytctp*^−IN+HA^ parasites on day 5 pi (Fig. 5i), suggesting an effect of the HA-tag on intron splicing and gene expression leading to changed asexual parasite growth.

**Fig. 6:**
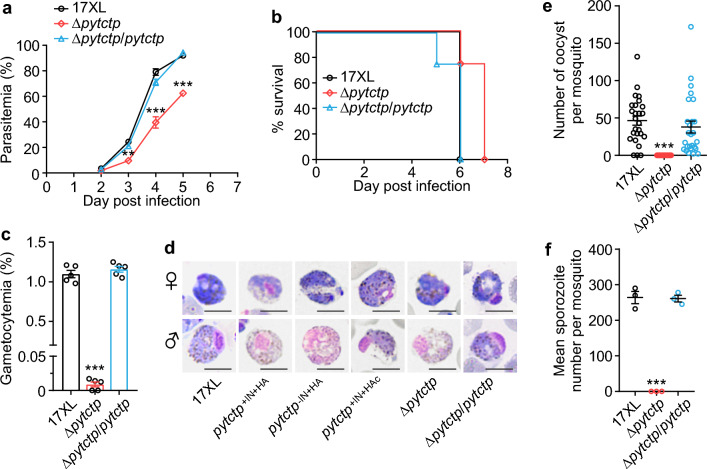
Parasitemia, host survival and counts of gametocyte, oocyst and sporozoite from parasites with *pytctp* gene deletion and complementation. **a** and **b** Parasitemia (**a**) and host survival (**b**) of 17XL wild type (WT), *pytctp* deleted and gene complemented parasites. **c** Gametocytemia of WT 17XL, *pytctp* deleted and gene-complemented parasites. **d** Images of Giemsa-stained male and female gametocytes of WT 17XL, *pytctp*-tagged, *pytctp*-deleted and gene-complemented parasites. The images were taken with a 100 × oil microscope objective. Scale bars, 5 μm. **e** and **f** Oocyst (**e**) and sporozoite counts (**f**) of WT 17XL, *pytctp* deleted and gene complemented parasites. Unpaired two-tailed *t*-test; **P < 0.01; ***P < 0.001

We also counted gametocytes in the blood of infected mice. The gametocyte rates of the parasite clones with 3xHA-tag (*pytctp*^+IN+HA^, *pytctp*^−IN+HA^ and *pytctp*^+IN+HAc^) were significantly lower than WT 17XL or the un-tagged clones without the intron (*pytctp*^−IN^), indicating that the introduction of 3xHA-tag in *pytctp* influenced the gametocyte production (Fig. 5j). No oocyte in the mosquito midgut or sporozoite in the salivary gland was observed for the parasite clones bearing 3xHA-tag in *pytctp* (Fig. 5k, l). We also generated a *pytctp* mutant parasite using the CRISPR/Cas9 technique and obtained a clonal parasite with the deletion of *pytctp* (∆*pytctp*, Fig. S3d-3f). *pytctp* deletion also led to slower asexual growth and severely defective gametocyte formation, and no oocyst or sporozoite was observed in the ∆*pytctp* parasite (Fig. 6a–f). We further re-introduced the *pytctp* gene into the ∆*pytctp* parasite to restore the gene function (Fig. S3d-3f). The rescued parasite (∆*pytctp*/*pytctp*) showed WT levels of asexual growth and production of gametocyte, oocyst and sporozoite (Fig. 6a–f). These results suggest that PyTCTP plays an essential role in the development of gametocyte and parasite transmission.

## Discussion

This study shows that 5’UTR introns of the malaria parasite *P. yoelii* play a role in gene expression regulation: The presence of a 5’UTR intron can generally increase mRNA and protein expression. As we are aware, this is the first demonstration of malaria UTR introns in regulating parasite gene expression, although the roles of UTR introns in gene expression have been reported in plants and other organisms [21–23]. Introns are expected to impose a substantial energetic burden on a cell, and the reasons for an organism to maintain a large number of introns have been issues of interest [16]. Here, we used two approaches to investigate the roles of 5’UTR introns in gene expression. We first used episomal plasmids carrying dual-luciferase expression cassettes to evaluate the effects of 5’UTR regions on the expression of four *P. yoelii* genes. Our results show significantly higher luciferase signals in parasites carrying 5’UTR introns than in those without introns in three of the four genes tested, suggesting that the presence of the introns improves the transcription and/or translation for some of the parasite genes.

To investigate the effect of 5’UTR introns on gene expression in an endogenous setting, we also used the CRISPR/Cas9 method to introduce a 3xHA-tag at 5’end of the gene encoding PyTCTP protein and generated parasites with or without 5’UTR intron at the same time. Analysis of protein expression using anti-HA antibody again showed significantly higher protein expression in the parasites with the 5’UTR intron. The episomal expression does not require the transport of mRNA out of the nuclear membrane; in this case, the 5’UTR introns do not contribute to mRNA export; therefore, the increase in gene expression could be through enhancement of transcription and/or translation processes. Alternative splicing (AS) coupled with nonsense-mediated decay (NMD) is an important post-transcriptional mechanism for regulating gene expression. The presence or absence of 5’ and 3’UTR may trigger AS-NMD of alternatively spliced mRNA. Upstream open reading frames overlapping the main start codon and long 3’UTRs (> 350 nt) can be the triggers for the AS-NMD mechanism. However, analysis of the 5’UTR sequences for the four *P. yoelii* genes we tested here did not show the presence of any new open reading frames overlapping the main start codon that may trigger AS-NMD in the sequences without introns. At this point, we do not know the exact mechanisms of how the introns enhance gene expression, which requires further investigation.

Our results also show that the strength of 5’UTR introns in enhancing gene expression depends on intron size, not the intron sequences. Two plasmid constructs carrying half of the original 5’UTR intron from the gene encoding PyTCTP protein had significantly lower luciferase signals than that of the parasite with the full-length intron. In contrast, plasmids carrying inversed DNA sequences of the original intron and a synthetic DNA random sequence with the same size as the full-length original intron did not significantly change gene expression levels, suggesting that the 5’UTR intron sequence may not act as a specific binding site for transcription factors. We recognize that these results were obtained from the 5’UTR intron of the *pytctp* gene, and the luciferase assays were performed in episomally transfected parasites. Experiments using additional genes and/or in vivo assays are necessary to draw a solid conclusion. Similar observations of size-dependent activity have been reported in plants; the longer the intron, the greater the relative level of gene expression. In humans, 5'UTR introns can also enhance the expression of some genes in a length-dependent manner, although the most highly expressed genes were found to have short 5'UTR introns [37]. Therefore, the mechanisms of 5’UTR-intron-mediated gene expression regulation are species-dependent. We still do not know how intron size influences gene expression. The presence of an intron with a specific size may create a unique space or secondary structure that promotes the binding of various transcription factors.

No significant difference was observed in the mRNA expression for the PY17X_0406400 (encoding a ubiquitin-conjugating enzyme E2) gene with or without a 5’UTR intron. The 5’UTR from the PY17X_0406400 had a low level of activity, possibly due to the relatively short 5’UTR that might miss some critical promoter elements in the cloned DNA segments. Attentively, sequence composition and GC content of the 5’UTR of this gene may play a role, which can be tested through modification of nucleotide sequences or replacement of its original sequence with 5’UTRs from other genes. There is also a possibility that not all the 5’UTRs can regulate gene expression.

We also introduced 5’UTR sequences with or without an intron from *P. y. yoelii* 17XL strain into *P. y. yoelii* BY265 and a subspecies of *P. y. nigeriensis* NSM. In both cases, the luciferase signals in BY265 and NSM parasites receiving promoter region without 5’UTR intron were significantly lower than those when plasmid with 5’UTR intron from *P. yoelii* 17XL was introduced into the same parasite strain. These results suggest that a similar mechanism operates in these parasite strains and that polymorphism in the 5’UTR regions (996 bp) may also affect gene expression, which can also be regulated through secondary structures.

We further investigated the biological effects of changes in the expression of the gene encoding PyTCTP. HA tagging of the gene at both N- and C-terminals appears to reduce parasite asexual growth rate for parasites with or without the 5’UTR intron but does not significantly affect host survival, suggesting an impact on parasite asexual growth by the HA-tagging of PyTCTP. A close examination of parasitemia also shows a significantly faster growth of the *pytctp*^+IN+HA^ parasites than the *pytctp*^−IN+HA^ day 5 pi, suggesting an effect of the 5’UTR intron on PyTCTP protein expression and parasite asexual growth. Therefore, the 5’UTR intron enhances PyTCTP expression and asexual growth, whereas the HA-tag hinders the development of both asexual and sexual stages. For the 17XL WT parasite, the 5’UTR intron is likely spliced in the asexual stages, and the WT and *pytctp*^−IN^ parasites would grow similarly (Figs. 4a and 5e). For the HA-tagged parasites, intron slicing and the stability of mRNA and/or protein could be affected by the HA-tag, leading to changed growth phenotypes.

Interestingly, both the *pytctp* gene with HA-tag and disruption of the gene (no expression) led to a significant reduction or absence in gametocytes, mature oocyst and sporozoites in the mosquitoes. The results suggest that PyTCTP also plays an essential role in the development of gametocyte and possibly mosquito stages. The N-terminal HA-tag did not significantly affect mRNA levels of PyTCTP in blood stages, and the PyTCTP protein was expressed at a reasonable level if the 5’UTR intron was present. However, the HA tagging significantly impaired parasite sexual development, which suggests that the HA-tag may disrupt the protein function. Intriguingly, previous studies showed that the homolog of the *Pb*ANKA *tctp* gene (*pbtctp*) was not essential, and deletion of the *pbtctp* did not significantly affect asexual parasite growth and host survival when mice were injected with iRBCs [38, 39]. However, significantly lower parasitemia and better survival were observed when mice were infected with sporozoites without the *pbtctp* gene. Additionally, similar numbers of infected mosquitoes and the numbers of sporozoites per infected mosquito salivary gland were found for the WT and *pbtctp* deficient parasites, suggesting that *pbtctp* is dispensable for *Pb*ANKA parasite development inside mosquitoes [38, 39]. How do we explain the differences observed between 17XL and *Pb*ANKA? The roles of *tctp* in the development of mosquito stages may be parasite species specific. Infections of *Anopheles stephensi* with *Pb*ANKA usually yield much higher oocyst counts than the oocyst numbers observed in this study (approximately 4 times the average oocyst count), and a reduction in oocyst number of *Pb*ANKA after the *pbtctp* deletion might not be evident if the numbers of oocysts were not counted precisely under controlled experimental conditions. Therefore, the phenotypic difference in mosquito stages between *Pb*ANKA and 17XL described here could be just a thresholding effect that results in few or no oocysts in the 17XL parasite but a milder phenotype in *Pb*ANKA. Another possibility is that the CRISPR/Cas9 procedures have off-target effects during the editing of the 17XL genome. However, possible off-target effects of the gRNAs can be ruled out after rescuing the *∆pytctp* with the wild-type allele, restoring infectivity to mosquitoes to wild-type levels. Additionally, three gRNAs were used in cutting the DNA sequences, two for HA tagging and one for gene deletion, and these experiments using different gRNAs led to similar defective phenotypes in sexual stages. The role of the *tctp* gene in the development of the mosquito stages between *Pb*ANKA and 17XL requires further investigation. If the essential role of TCTP in sexual stage development can be confirmed in other *Plasmodium* species, TCTP can be a potential target for developing drugs or vaccines to block parasite transmission.

## Conclusions

This study demonstrates that *Plasmodium* 5’UTR introns can enhance gene expression in a size-dependent manner; the presence of alternatively spliced mRNAs in different parasite developmental stages suggests that alternative slicing of 5’UTR introns is one of the key mechanisms in regulating parasite gene expression and differentiation.

### Supplementary Information

Supplementary Materials 1: Fig. S1 Nucleotide sequence alignment of *pytctp *5’UTR region. The sequences of *Plasmodium yoelii* 17XL were downloaded from PlasmoDB (https://plasmodb.org/); the sequences of NSM and BY265 strains were obtained in this study. The sequences were aligned using Clustal Omega (https://www.ebi.ac.uk/Tools/msa/clustalo/).

Supplementary Materials 2: Fig. S2 Generation of parasites with N-terminal HA-tagged *pytctp* gene driven by promoters with or without a 5’UTR intron. a and b: Diagrams showing strategies of CRISPR/Cas9-mediated knock-in to introduce sequence encoding 3xHA tag as generation of promoter regions with (a) or without (b) a 5’UTR intron. The arrows for F1, F2, F3, R1, R2 and R3 indicate the positions and directions of primers used in the experiments. c and d: Agarose gels of PCR products using primer pairs indicated in a and b (see Table S1 for primer sequences), showing parasite clones with expected PCR products. e and f: Electropherograms of DNA sequences confirming correct 3xHA tagging and the knock-in sequences.

Supplementary Materials 3: Fig. S3 Generation of parasites with C-terminal HA-tagged *pytctp* gene and *pytctp* gene deletion and re-introduction. a: A diagram showing CRISPR/Cas9-mediated insertion of an HA-tag into the *pytctp* gene at C-terminal. The arrows for F6, F4, R4 and R6 indicate the positions and directions of primers used in the experiments. b: An agarose gel showing parasite clones with expected PCR products using primer pairs indicated in a and Table S1. c: Electropherograms of DNA sequences confirming correct HA-tag insertion. d: A diagram showing CRISPR/Cas9-mediated disruption and re-introduction of the *pytctp* gene. The arrows for F5, R5, kF, mF, kR and mR indicate the positions and directions of primers used in the experiments. e: An agarose gel showing parasite clones with expected PCR products using primer pairs indicated in (d) and Table S1. e: Electropherograms of DNA sequences confirming gene disruption and in-frame re-introduction of deleted *pytctp* coding fragment.

Supplementary Materials 4: Table S1 Sequences of primers used in this study.

## Data Availability

All data generated or analyzed during this study are included in this article.
