# Supplementary material for: Size-dependent enhancement of gene expression by Plasmodium 5’UTR introns
Source: Parasit Vectors. 2024 May 27;17:238. doi: 10.1186/s13071-024-06319-0 (PMC11131223; doi:10.1186/s13071-024-06319-0)
Supplement: Supplementary file 1 — Supplementary Materials 1: Fig. S1 Nucleotide sequence alignment of pytctp 5’UTR region. The sequences of Plasmodium yoelii 17XL were downloaded from PlasmoDB (https://plasmodb.org/); the sequences of NSM and BY265 strains were obtained in this study. The sequences were aligned using Clustal Omega (https://www.ebi.ac.uk/Tools/msa/clustalo/). [file 13071_2024_6319_MOESM1_ESM.pdf]

```
NSM      TAGAGAATGCATTTTGTCATGTATTTCATTTTTAAGCACATTATTATTTGTTTCTGTAAT   60
17XL     TAGAGAATGCATTTTGTCATGTATTTCATTTTTAAGCACATTATTATTTGTTTCTGTAAT   60
BY265    TAGAGAATGCATTTTGTCATGTATTTCATTTTTAAGCACATTATTATTTGTTTCTGTAAT   60
         ************************************************************

NSM      ATTTAATTCATCGTTTTGTCTTTATAATAATAGCATAAGTATATGTTTATATTATACTTT   120
17XL     ATTTAATTCATCGTTTTGTCTTTATAATAATAGCATAAGTATATGTTTATATTATACTTT   120
BY265    ATTTAATTCATCGTTTTGTCTTTATAATAATAGCATAAGTATATGTTTATATTATACTTT   120
         ************************************************************

NSM      GAAGTGTCCAAGATATATATATATATATAATTACAAGCTTATTTTAATAATTATAGTATACT   180
17XL     GAAGTGTCCAAGATATAT--ATATATAATTACAAGCTTATTTTAATAATTATAGTATAGT   178
BY265    GAAGTGTCCAAGATATATATATATATATAATTACAAGCTTATTTTAATAATTATAGTATAGT   180
         ******************  ************************************** *

NSM      ATACTATACTAAATTGTACATAAAAAACACAA----------AAAATATACAATTTTTTA   230
17XL     ATAGTATACTAAATTGTACATAAAAAACACAAAAAACACAAAAAAATATACAATTTTTTA   238
BY265    ATAGTATACTAAATTGTACATAAAAAACACAAAAAACACAAAAAAATATACAATTTTTTA   240
         *** ****************************          ******************

NSM      AAAAGAGGCAATCTTATTAAATATTACACAGAAGGATTTTCCCCTTATAAAATATGTGAT   290
17XL     AAAAGAGGCAATCTTATTAAATATTACACAGAAGGATTTTCCCCTTATAAAATATGTGAT   298
BY265    AAAAGAGGTAATCTTATTAAATATTACACAGAAGGATTTTCCCCTTATAAAATATGTGAT   300
         ******** ***************************************************

NSM      ATAAATTAATATATCTTAGTTCCTGTATAATATACATACATTACTTATATTTTTAAGTAG   350
17XL     ATAAATTAATATATCTTAGTTCCTGTATAATATACATACATTACTTATATTTTTAAGTAG   358
BY265    ATAAATTAATATATCTTAGTTCCTGTATAATATACATACATTACTTATATTTTTAAGTAG   360
         ************************************************************

NSM      AACATTTTTGTTTCTTTTTATATCAATAAAAAACGATACAAACATATTTATTCACTCATT   410
17XL     AACATTTTTGTTTCTTTTTATATCAATAAAAAACGATACAAACATATTTATTCACTCATT   418
BY265    AACATTTTTGTTTCTTTTTATATCAATAAAAAACGATACAAACATATTTATTCACTCATT   420
         ************************************************************

NSM      TAATAATAATCCTTTGAAATTTTATGAATTTATATTAATTAGAATTAAATGCAATATATA   470
17XL     TAATAATAATCCTTTGAAATTTTATGAATTTATATTAATTAGAATTAAATGCAATATATA   478
BY265    TAATAATAATCCTTTGAAATTTTATGAATTTATATTAATTAGAATTAAATGCAATATATA   480
         ************************************************************

NSM      TAATAATAAAGAAAGAAATTTATTAAGCGCATTATTACCGTTGTCAAATATGCATTAAAA   530
17XL     TAATAATAAAGAAAGAAATTTATTAAGCGCATTATTACCGTTGTCAAATATGCATTAAAA   538
BY265    TAATAATAAAGAAAGAAATTTATTAAGCGCATTATTACCGTTGTCAAATATGCATTAAAA   540
         ************************************************************
```

```
NSM      AAAATAGTAATTATTATTTTATAAGCACACTTATTTATATATATATATATATCCGTAAAT   590
17XL     AAAATAGTAATTATTTTA---TAAGCACACTTATT--TATATATATATATATCCGTAAAT   593
BY265    AAAATAGTAATTATTTTA---TAAGCACACTTATA--TATATATATATATATCCGTAAAT   595
         *************** *    *************   ***********************

NSM      AGTAACATAATATATTTATTTTTTTT----ATTCATTGAATTTTTTATATTTGTTTCTTA   646
17XL     AGTAACATAATATATTTATTTTTTTTATTAATTCATTGAATTTTTTATATTTGTTTCTTA   653
BY265    AGTAACATAATATATTTATTTTTTTT----ATTCATTGAATTTTTTATATTTGTTTCTTA   651
         **************************    ******************************

NSM      TGTTAAAATAATATATTTTTATAA-TTTTTTTTTTAAGCTTAAGATCGTTTATCTTTAAA   705
17XL     TGTTAAAATAATATATTTTTATAAATTTTTTTTTTAAGCTTAAGATCGTTTATCTTTAAA   713
BY265    TGTTAAAATAATATATTTTTATAAATTTTTTTTTTAAGCTTAAGATCGTTTATCTTTAAA   711
         ************************ ***********************************

NSM      ACTTTGgtaaatataaatataaaaaattatagcataatattatatatattaaaagtgaaa   765
17XL     ACTTTGgtaaatataaatataaaaaattatagcataatattatatatattaaaagtgaaa   773
BY265    ACTTTGgtaaatataaatataaaaaattatagcataatattatatatattaaaagtgaaa   771
         ************************************************************

NSM      aacttaaaagcatgcatataatataatatatcgatatcccctttttatttttttgtgataa   825
17XL     aacttaaaagcatgcatataatataatatatcgatatcccctttttatttttttgtgataa   833
BY265    aacttaaaagcatgcatataatataatatatcgatatcccctttttatttttttgtgataa   831
         ************************************************************

NSM      tattaaatcataaattcatatttttcttctttctttttagAAGCCTTTTAATCATATTGT   885
17XL     tataaaatcggaaattcatatttttcttctttctttttagAAGCCTTTTAATCATATTGT   893
BY265    tattaaatcagaaattcatatttttcttctttctttttagAAGCCTTTTAATCATATTGT   891
         *** *****  *************************************************

NSM      ATTTTTAAGGAAACTAGTTGTAATTTTTAATTATAAGGATATTTACTGCTATATTTACGT   954
17XL     ATTTTTAAGGAAACTAGTTGTAATTTTTAATTACAAGGATATTTACTGCTATATTTACGT   953
BY265    ATTTTTAAGGAAACTAGTTGTAATTTTTAATTACAAGGATATTTACTGCTATATTTACGT   951
         ********************************* **************************

NSM      GTATAAACAAATATTATTTTGTTCGATATTTGTATAAGCCAAA   988
17XL     GTATAAAAAAATATTATTTTGTTCGATATTTGTATAAGCCAAA   996
BY265    GTATAAAAAAATATTATTTTGTTCGATATTTGTATAAGCCAAA   994
         ******* ***********************************
```
