# Supplementary figures and images for: Size-dependent enhancement of gene expression by Plasmodium 5’UTR introns
Source: Parasit Vectors. 2024 May 27;17:238. doi: 10.1186/s13071-024-06319-0 (PMC11131223; doi:10.1186/s13071-024-06319-0)

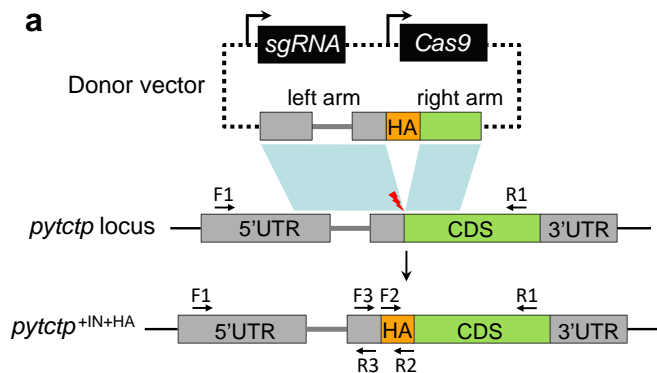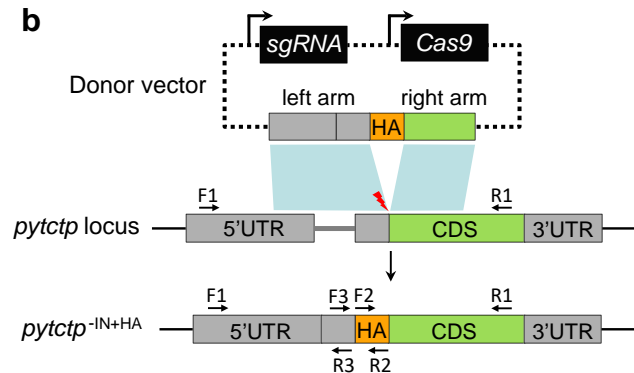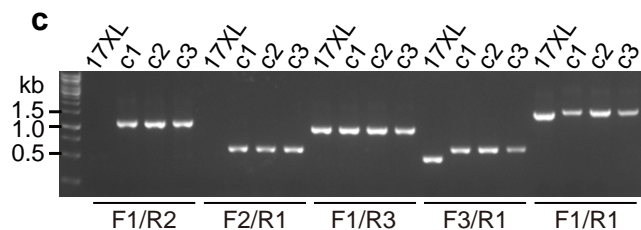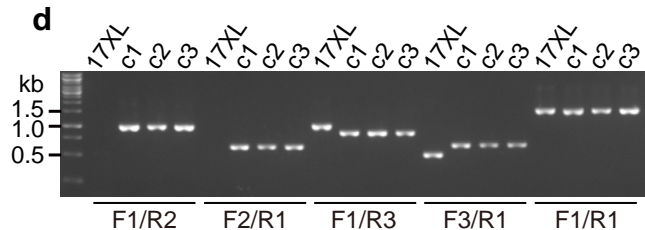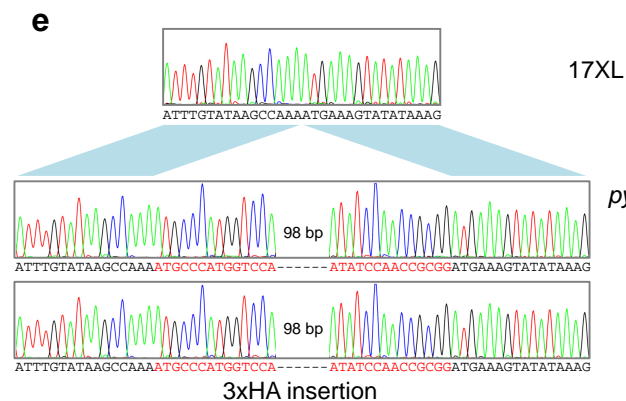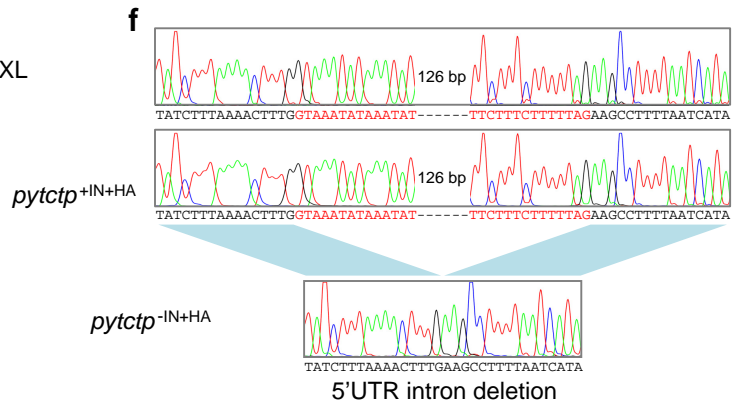

Supplement: Supplementary file 2 — Supplementary Materials 2: Fig. S2 Generation of parasites with N-terminal HA-tagged pytctp gene driven by promoters with or without a 5’UTR intron. a and b: Diagrams showing strategies of CRISPR/Cas9-mediated knock-in to introduce sequence encoding 3xHA tag as generation of promoter regions with (a) or without (b) a 5’UTR intron. The arrows for F1, F2, F3, R1, R2 and R3 indicate the positions and directions of primers used in the experiments. c and d: Agarose gels of PCR products using primer pairs indicated in a and b (see Table S1 for primer sequences), showing parasite clones with expected PCR products. e and f: Electropherograms of DNA sequences confirming correct 3xHA tagging and the knock-in sequences. [file 13071_2024_6319_MOESM2_ESM.pdf]

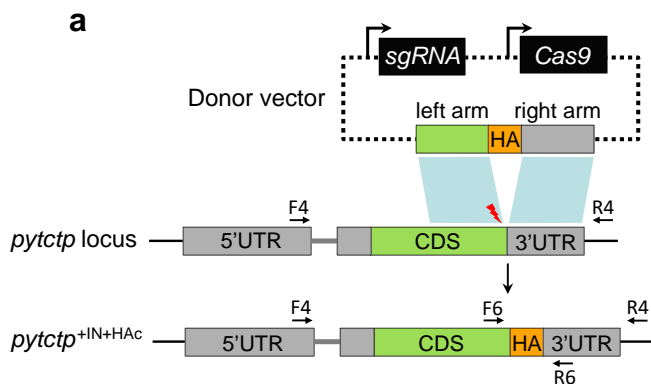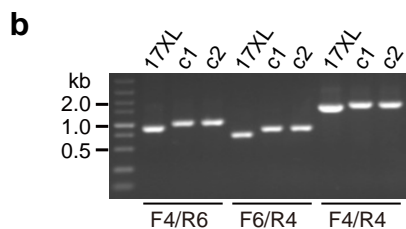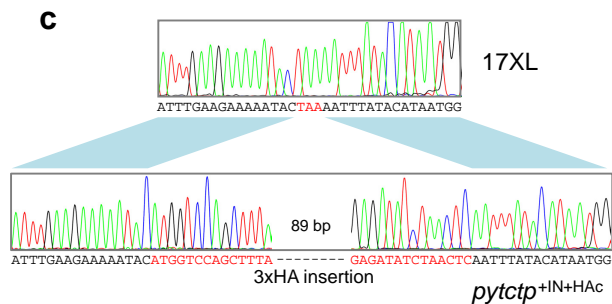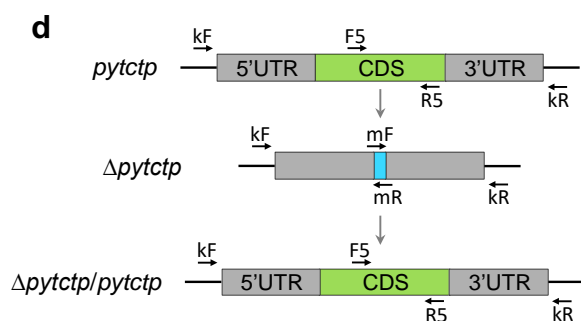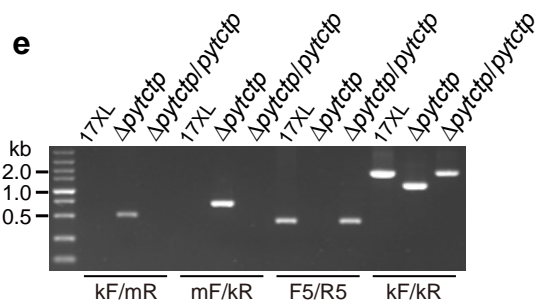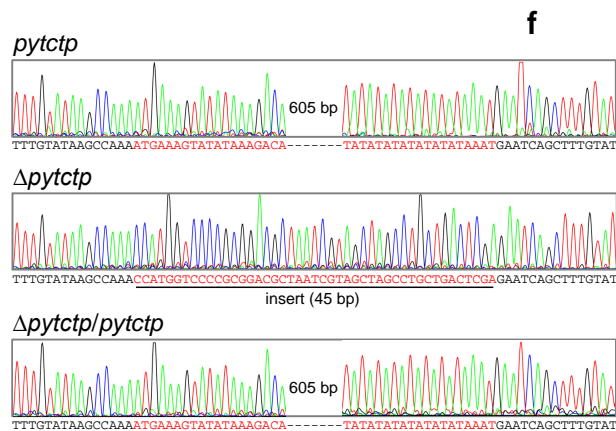

Supplement: Supplementary file 3 — Supplementary Materials 3: Fig. S3 Generation of parasites with C-terminal HA-tagged pytctp gene and pytctp gene deletion and re-introduction. a: A diagram showing CRISPR/Cas9-mediated insertion of an HA-tag into the pytctp gene at C-terminal. The arrows for F6, F4, R4 and R6 indicate the positions and directions of primers used in the experiments. b: An agarose gel showing parasite clones with expected PCR products using primer pairs indicated in a and Table S1. c: Electropherograms of DNA sequences confirming correct HA-tag insertion. d: A diagram showing CRISPR/Cas9-mediated disruption and re-introduction of the pytctp gene. The arrows for F5, R5, kF, mF, kR and mR indicate the positions and directions of primers used in the experiments. e: An agarose gel showing parasite clones with expected PCR products using primer pairs indicated in (d) and Table S1. e: Electropherograms of DNA sequences confirming gene disruption and in-frame re-introduction of deleted pytctp coding fragment. [file 13071_2024_6319_MOESM3_ESM.pdf]
